# Causal association between cardiovascular diseases and erectile dysfunction, a Mendelian randomization study

**DOI:** 10.3389/fcvm.2023.1094330

**Published:** 2023-02-09

**Authors:** Qingying Li, Qiang Long, Baoming Ren, Sen Bing

**Affiliations:** ^1^Department of Urology, Xi’an No.3 Hospital, The Affiliated Hospital of Northwest University, Xi’an, Shanxi, China; ^2^Department of Cardiology, Xi’an No.3 Hospital, The Affiliated Hospital of Northwest University, Xi’an, Shanxi, China

**Keywords:** cardiovascular disease increases the risks of erectile dysfunction atrial fibrillation, coronary heart disease, erectile dysfunction, heart failure, ischemic heart disease, Mendelian randomization

## Abstract

**Background:**

Cardiovascular diseases (CVD), including coronary heart disease (CHD), heart failure, ischemic heart disease (IHD), and atrial fibrillation, are prevalent in the aged. However, the influence of CVD on ED is less investigated. This study was performed to clarify the causal association between CVD and ED.

**Materials and methods:**

Genome-wide association studies (GWAS) datasets targeting CHD, heart failure, IHD, and atrial fibrillation were downloaded to retrieve single nucleotide polymorphisms (SNPs). Further, single-variable Mendelian randomization and multivariable Mendelian randomization (MVMR) were adopted to explore the causal association between CVD and ED.

**Results:**

Genetically predicted CHD and heart failure were found to increase the risks of ED (OR = 1.09, *P* < 0.05 and OR = 1.36, *P* < 0.05, respectively). However, no causal association was disclosed among IHD, atrial fibrillation and ED (all *P* > 0.05). These findings remained consistent in sensitivity analyses. After controlling for body mass index, alcohol, low density lipoprotein, smoking and total cholesterol levels, the results of MVMR support the causal role of CHD on ED (*P* < 0.05). Similarly, the direct causal effect estimates of heart failure on ED were significant in MVMR analyses (*P* < 0.05).

**Conclusion:**

Using genetic data, this study revealed that genetically predicted CHD and heart failure may predict better ED compared with atrial fibrillation and IHD. The results should be interpreted with caution and the insignificant causal inference of IHD still needs further verification in future studies.

## 1. Introduction

Erectile dysfunction (ED) is characterized by the failure of penile erection to complete satisfactory sexual intercourse (1). As reported by Yang et al. (2), anxiety and depression occur in 79.82 and 79.56% of patients with ED, respectively. In light of the currently high ED prevalence, reducing the occurrence of ED is requisite. Therefore, it is important to investigate better on the biological basis of ED, which may be related to a genetic predisposition background in common to that of cardiovascular diseases (CVD).

Aside from the widely accepted risk factors including cigarette and alcohol consumption, diabetes, sleep disorders, and obstructive sleep apnea syndrome, evidence about the causal association between CVD and ED is limited (2–4). ED often precedes a CVD event by 2–5 years. One umbrella review by Mostafaei et al. (5) disclosed that patients with ED have a higher risk of CVD, coronary heart disease (CHD), cardiovascular-related mortality, all-cause mortality, and myocardial infarction. Some other studies also reported that ED is an early indicator for CVD (6). Given the mortality of CVD, patients with ED are recommended to receive cardiological evaluation in clinical practice. Endothelial dysfunction may be the common origin at the early onset of the two diseases. Therefore, it is necessary to clarify their causal association in clinical settings. However, the majority of previous studies relied on observational designs, which cannot avoid the biases from confounders and reverse causality, and further clarify the causal association between ED and CVD. Thus, as opposed to previous reports, Tian et al. (7) found that ED is not associated with an increased risk of CHD in a Chinese Han population. Likewise, in the Netherlands, Speel et al. (8) observed an insignificant association in participants aged 40–50 and 60–70 years. Additionally, other pathogeneses such as genetic cardiac ion channel dysfunction may lead to CVD first. However, the further influence of CVD, including CHD, heart failure, ischemic heart disease (IHD) and atrial fibrillation, on ED is rarely investigated. The causal association between cardiovascular events and ED remains weak and unclear.

To address the confounders and reverse causality between CVD and ED, we adopted a novel framework, Mendelian randomization (MR) to explore their causal links. MR extracts the single nucleotide polymorphisms (SNPs) significantly associated with exposure (i.e., CHD, IHD, atrial fibrillation) as instrumental variables (IVs) (9). These SNPs are randomly assorted when forming zygotes, indicating the random distribution of genetic instruments. Supposing that SNPs are unevenly distributed in the outcomes (i.e., ED), a possible causal direction from exposure to outcomes is established. In this study, using genome-wide association study (GWAS) datasets from previous studies, we performed single variable MR (SVMR) and multivariable MR (MVMR) to assess the causal association between CVD (CHD, heart failure, IHD, and atrial fibrillation) and ED. Clear causal links between them may be conducive to clinical decision-making.

## 2. Materials and methods

### 2.1. Data sources and study samples

To perform two-sample MR analyses, five summary-level GWAS datasets were used. Datasets regarding CHD (10, 11), heart failure (12, 13), IHD (Neale LabGWAS round 2),^1^ atrial fibrillation (14), and ED (15) were retrieved from five previous cohorts.

The summary-level dataset of CHD was obtained from the Coronary Artery Disease Genome-Wide Replication and Meta-analysis plus the Coronary Artery Disease Genetics (CARDIoGRAMplusC4D) consortium (10). This database meta-analyzed 48 studies and included 184,305 participants (60,801 cases and 123,504 controls). Among them, 77% subjects were of European ancestry, and 19% participants were of Asian ancestry. The diagnosis of coronary artery disease was defined as myocardial infarction, acute coronary syndrome, chronic stable angina pectoris, or coronary artery stenosis (>50%) (11).

The genetic association of heart failure was calculated by the Heart failure molecular epidemiology for therapeutic targets (HERMES) consortium (12, 13). The diagnosis of heart failure was based on clinical diagnosis for any etiology, but without objective evidence from the left ventricular ejection fraction. By combining 26 cohorts from the European ancestry, 47,309 cases and 930,014 controls were pooled and subjected to further analyses. In their study, variants, whose imputation quality was <0.5, or MAF was <0.01, or absolute betas and standard error were >10, were removed. Finally, 12 independent variants at 11 genomic loci were identified to be significantly associated with heart failure.

Another GWAS dataset from the Neale Lab (see text footnote 1; GWAS round 2) investigated the genetic loci associated with IHD. Using data from the UK biobank, this study included 361,194 European individuals (20,857 IHD cases and 340,337 controls). The diagnosis of IHD mainly relied on the codes of International Classification of Diseases version 10 (ICD-10: I25.9, I24, and I25).

The summary-level dataset of atrial fibrillation was downloaded from one previous GWAS by Jonas et al. (14). This GWAS enrolled 1,030,836 participants (60,620 cases and 970,216 controls) from six studies (The Nord-Trøndelag Health Study, deCODE, the Michigan Genomics Initiative, DiscovEHR, UK Biobank, and the AFGen Consortium). All the participants received an electrocardiogram examination and were then evaluated by artificial algorithm to distinguish the atrial fibrillation and normal sinus rhythm participants. This study identified 111 atrial fibrillation-associated loci.

The SNPs closely associated with ED were obtained from one GWAS by Jonas et al. (15). A total of 223,805 participants of European ancestry were included (6,175 ED cases versus 217,630 controls). The diagnosis of ED was based on ICD-10 codes (N48.4 and F52.2) and medical histories of drug or surgical intervention in ED. In total, 9,310,196 SNPs were detected in their cohort and used as the outcome for harmonization in this MR study.

The genetic data of the adjusted covariates including body mass index (BMI), cigarette and alcohol consumption, low-density lipoprotein (LDL), and total cholesterol levels were retrieved from previous GWAS (16, 17). The enrolled participants were all of the European ancestry to avoid population architecture bias. Detailed information on the definition, genotyping, quality control and imputation can be accessed in the methods section or the Supplementary files of the original articles.

### 2.2. MR assumptions and genetic instrument selection

The causal association can be inferred based on satisfaction of three basic MR assumptions: (1) strongly associated with exposures (relevance assumption); (2) not associated with confounders of the association between exposures and ED (independence assumption), and (3) the association with ED risk was only found *via* exposures (exclusivity assumption). Only when the three basic MR assumptions are satisfied, then the further causal association can be inferred. Previous GWASs have identified the genetic correlation between IVs and exposures to satisfy the relevance assumption, which was set as *P* < 5 × 10^–8^ in our study. The qualified IVs are used to replace the exposures (i.e., CHD) and the outcome (i.e., ED), which are not influenced by the postnatal factors like obesity. Therefore, a possible causal association between exposure and outcome is established.

The SNPs extracted from exposures (CHD, heart failure, stroke, IHD, and atrial fibrillation) were filtered by the following criteria: *P* < 5 × 10^–8^ and linkage disequilibrium *r*^2^ < 0.01 at a window size of 1 Mb. Further, the MR-Steiger method was performed on the left SNPs to calculate the variance explained by the exposure and outcome (18). The insignificant results of MR-Steiger indicated that the left SNPs may affect the outcome (i.e., ED) more than the exposure (i.e., CHD). Additionally, to avoid bias from weak IVs, F-statistics of SNPs were calculated using the following formula: F-statistics = (Beta/Se)^2^ (3). The values of F-statistics represented the strength of IVs, and generally, F-statistics < 10 were accepted as weak IVs. Finally, 43, 9, 31, and 139 SNPs were selected as IVs for CHD, heart failure, IHD, and atrial fibrillation, respectively. The IVs are shown in Supplementary Tables 1–4.

### 2.3. Detection of heterogeneity and pleiotropy

Cochran’s Q test was applied to evaluate the heterogeneity between the genetic instruments. Three methods including MR-Egger regression intercept, MR Pleiotropy Residual Sum and Outlier (MR-PRESSO), and radial MR were used to detect the pleiotropic outliers (19, 20). The identified pleiotropic outliers were removed before harmonization and further MR analyses. This endeavor ensures that the IVs meet the three basic assumptions of MR in statistics.

### 2.4. Sensitivity analyses

The inverse-variance-weighting (IVW) approach was used to test the causal effect estimates between the cardiovascular risk factors and ED. A false discovery rate (FDR) was used to adjust for multiple testing. To verify the robustness of the conclusion, we further adopted several different methods including MR-Egger, Weighted median, Maximum likelihood, robust adjusted profile score (MR.RAPS) and MR-PRESSO. The MR-Egger approach introduces an intercept term into the Egger regression model (19). The distance from intercept item to 0 can be used to detect the presence of directional pleiotropy. Even when all the IVs are invalid, this method can still produce valid causal estimates. Compared with the MR-Egger method, the weighted median estimator can identify causal effects even if half of the IVs are invalid (3). We also adopted the maximum likelihood estimator to verify the findings. Despite having low standard errors, the maximum likelihood method may be biased by small sample sizes (21). The MR.RAPS approach was developed by Zhao et al. (22). This estimator can yield consistent causal estimates when weak and pleiotropic IVs exist. Likewise, the MR-PRESSO estimator can produce robust causal estimates when pleiotropy exists. MR-PRESSO is optimally suited when 50% of IVs exhibit horizontal pleiotropy (20). These approaches show different statistical power in different scenarios.

### 2.5. Multivariate Mendelian randomization analyses

Aside from the total effects of cardiovascular risk factors on ED, the direct effects of these risk factors can be obtained using MVMR, adjusting for possible confounders. Given that CVDs are often accompanied with poor living habits and possibly metabolic disorders, we further adjusted the BMI, cigarette and alcohol consumption, LDL, and total cholesterol levels. MVMR analyses used the overlapping SNPs between exposures and cofounders as IVs, which were then meta-analyzed using the IVW approach (3).

All analyses and figures in this study were made using R 4.0.2 software (R Foundation for Statistical Computing, Vienna, Austria). In different analyses, packages including “TwoSampleMR,” “forestplot,” and “RadialMR” were used. In statistics, *P* < 0.05 was considered significant.

## 3. Results

### 3.1. Causal effect estimates of genetically predicted CVDs on ED by the IVW estimator

**FIGURE 1:**
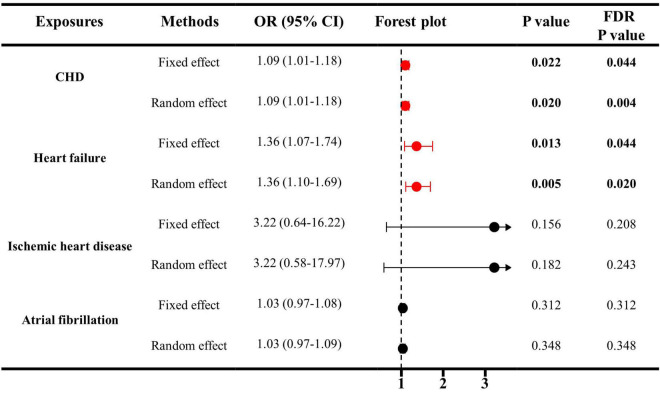
The causal effects of cardiovascular diseases on ED by IVW estimator. IVW, inverse variance weighting; CHD, cardiovascular diseases; ED, erectile dysfunction; SNP, single nucleotide polymorphisms; OR, odds ratio; CI, confidence interval.

**FIGURE 2:**
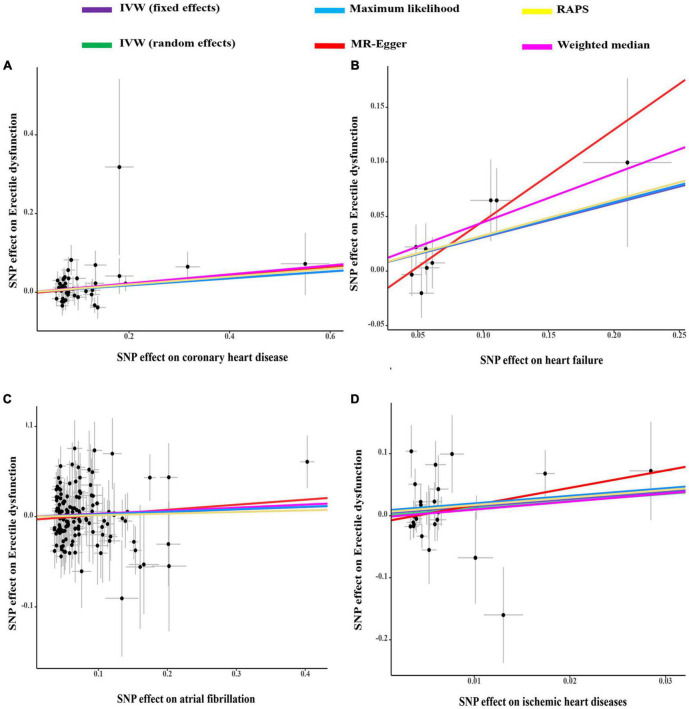
The scatter plots of coronary heart disease **(A)**, heart failure **(B)**, atrial fibrillation **(C)**, and ischemic heart disease **(D)** on ED. IVW, inverse variance weighting; MR, Mendelian randomization; SNP, single nucleotide polymorphisms; ED, erectile dysfunction.

As indicated in Figure 1, genetically predicted CHD and heart failure were found to increase the risks of ED (OR = 1.09, *P* < 0.05 and OR = 1.36, *P* < 0.05). However, no causal association was disclosed between IHD (OR = 3.22, *P* > 0.05), atrial fibrillation (OR = 1.03, *P* > 0.05) and ED. In Figures 2A, B, the scatter plot disclosed that the SNP effect on ED increased with the increase of the SNP effect on CHD and heart failure. In Figures 2C, D, this increasing trend was also noted for IHD, but it did not reach the significant threshold as revealed by Figure 1.

In the SVMR analyses, no signs of weak IVs, and pleiotropy were detected. Table 1 shows that the average F-statistics were all greater than 10, indicating the minimal likelihood of bias from weak IVs. Moreover, the Global tests, MR-Egger regression intercepts and radial MR method identified no pleiotropic outliers (all *P* > 0.05), suggesting the absence of pleiotropy. As for heterogeneity (Table 2 and Supplementary Figure 1), the results of Cochran’s Q tests were not significant (all *P* > 0.05 by the MR-Egger, IVW, and maximum likelihood methods). Therefore, these data did not support the presence of heterogeneity.

**TABLE 1:** Characteristics of included datasets.

Trait	nSNP	F statistics	Sample size	Consortium	Descent	PMID	Unit
Coronary artery disease	43	59.78	184,305 (60,801 cases and 123,504 controls)	CARDIoGRAMplusC4D	77% European 19% Asian ancestry	26343387	logOR
Heart failure	9	42.04	977,323 (47,309 cases and 930,014 controls)	HERMES	European	31919418	logOR
Ischemic heart disease	31	65.34	361,194 (20,857 IHD cases and 340,337 controls)	Neale Lab	European	NA	logOR
Atrial fibrillation	139	84.35	1,030,836 (60,620 cases and 970,216 controls)	NA	European	30061737	logOR
Erectile dysfunction	–	–	223,805 (6,175 cases and 217,630 controls)	NA	European	30583798	logOR

SNP, single nucleotide polymorphisms; PMID, PubMed identifier; OR, odds ratio; CARDIoGRAMplusC4D, Coronary Artery Disease Genome-Wide Replication and Meta-analysis plus the Coronary Artery Disease Genetics; HERMES, Heart Failure Molecular Epidemiology for Therapeutic Targets; NA, not available.

**TABLE 2:** Results of heterogeneity and pleiotropy tests.

Exposures	*P-*values of global test	MR-egger intercept (*P*-value)	Tests of heterogeneity		
			***P-*value of MR-egger**	***P-*value of IVW**	***P-*value of ML**
CHD	0.508	−0.006 (0.610)	0.499	0.539	0.541
Heart failure	0.635	−0.038 (0.128)	0.869	0.630	0.641
Ischemic heart disease	0.297	−0.011 (0.323)	0.287	0.285	0.285
Atrial fibrillation	0.110	−0.001 (0.396)	0.274	0.279	0.279

CHD, coronary heart disease; IVs, instrumental variables; IVW, inverse variance weighting; MR, Mendelian randomization; ML, maximum likelihood.

### 3.2. Sensitivity analysis of causality between genetically predicted CVDs and ED

**FIGURE 3:**
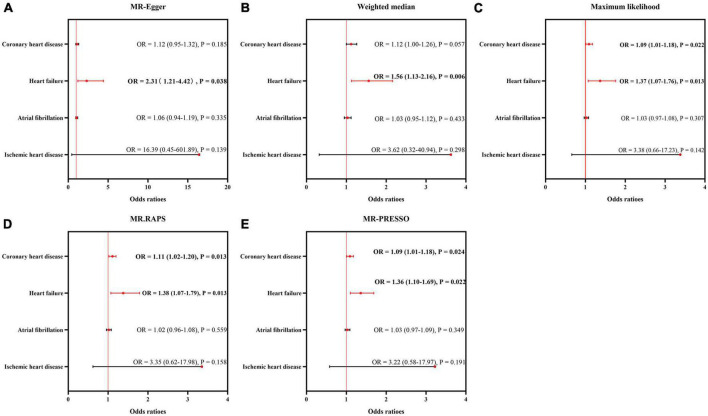
Sensitivity analysis for the causality of cardiovascular diseases on ED. The results of MR-Egger **(A)**, weighted median **(B)**, maximum likelihood **(C)**, MR.RAPS **(D)**, and MR-PRESSO **(E)**. MR, Mendelian randomization; MR-PRESSO, MR Pleiotropy Residual Sum and Outlier; MR.RAPS, robust adjusted profile score; OR, odds ratio.

The results of sensitivity analyses using five approaches are displayed in Figure 3. The sizes and significance of causal estimates between CHD and ED remained consistent in the Maximum likelihood, MR.RAPS and MR-PRESSO estimators (OR = 1.09, *P* < 0.05; OR = 1.11, *P* < 0.05; and OR = 1.09, *P* < 0.05, respectively). Although similar in sizes of causal effects (OR = 1.12 for MR-Egger and Weighted median), the significance was not replicated by the MR-Egger and Weighted median methods (*P* = 0.185 for MR-Egger and *P* = 0.057 for the Weighted median), suggesting limited effectiveness.

Additionally, the sizes and significance of causal estimates between heart failure and ED remained consistent in all the five approaches (MR-Egger: OR = 2.31, *P* < 0.05; Weighted median: OR = 1.56, *P* < 0.05; Maximum likelihood: OR = 1.37, *P* < 0.05; MR.RAPS: OR = 1.38, *P* < 0.05; MR-PRESSO: OR = 1.36, *P* < 0.05). These data supported the risky role of heart failure in the suffering of ED.

Similar to the IVW estimator, IHD, and atrial fibrillation were not causally associated with the occurrence of ED by the MR-Egger, weighted median, maximum likelihood, MR.RAPS and MR-PRESSO methods (all *P* > 0.05).

### 3.3. Direct causal effect estimates of genetically predicted CVDs on ED in MVMR

**FIGURE 4:**
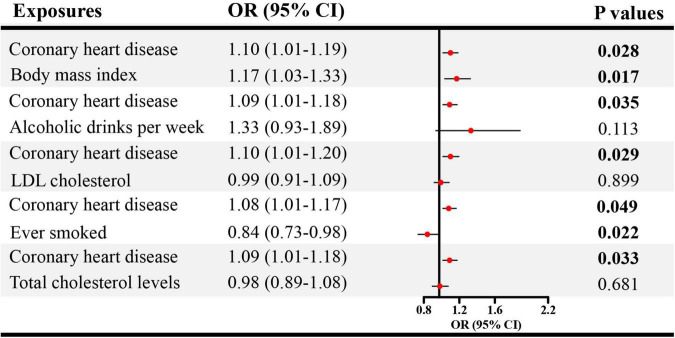
The direct causal effects of CHD on ED in MVMR. CHD, coronary heart disease; MVMR, multivariable Mendelian randomization; OR, odds ratio; CI, confidence interval.

**FIGURE 5:**
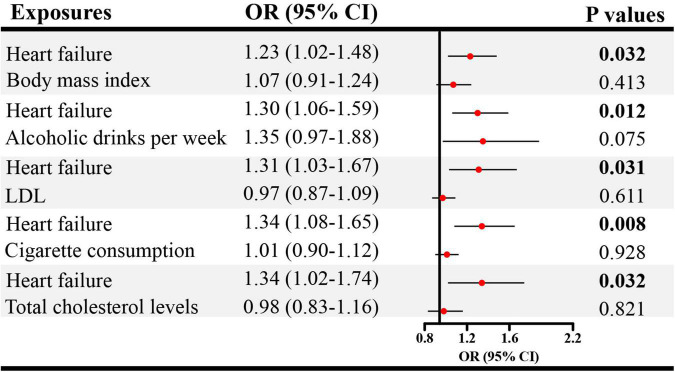
The direct causal effects of heart failure on ED in MVMR. MVMR, multivariable Mendelian randomization; OR, odds ratio; CI, confidence interval.

Given the significant causal association between CHD, heart failure and ED in SVMR, further MVMR was applied. In Figure 4, the direct causal effect estimates of CHD on ED were 1.10 (95% CI = 1.01–1.19, *P* < 0.05), 1.09 (95% CI = 1.01–1.18, *P* < 0.05), 1.10 (95% CI = 1.01–1.20, *P* < 0.05), 1.08 (95% CI = 1.01–1.17, *P* < 0.05), and 1.09 (95% CI = 1.01–1.18, *P* < 0.05), after controlling for BMI, alcohol, LDL, cigarette and total cholesterol levels, respectively. Besides, in Figure 5 the direct causal effect estimates of heart failure on ED were 1.23 (95% CI = 1.02–1.48, *P* < 0.05), 1.30 (95% CI = 1.06–1.59, *P* < 0.05), 1.31 (95% CI = 1.03–1.67, *P* < 0.05), 1.34 (95% CI = 1.08–1.65, *P* < 0.01), and 1.34 (95% CI = 1.02–1.74, *P* < 0.05), after controlling for BMI, alcohol, LDL, cigarette and total cholesterol levels, respectively.

## 4. Discussion

This two-sample MR study used summary-level data from previous GWAS to explore the causal links between CVD and ED. On the basis of genetic data, this study avoids confounding bias, selection bias, and reverse causality, in observational surveys. The principal finding of this study is that genetically predicted CHD and heart failure may predict better ED as compared with atrial fibrillation and IHD. These findings warn against the ED risk of patients with CHD and heart failure. Therefore, screening and evaluation of erectile function should be considered for populations at high risk of CHD and heart failure.

The association between CVDs and erectile function has been frequently investigated in previous studies. As reported by Ma et al. (23), a 1.58-fold increased risk of CHD was observed for patients with ED during a median follow-up of 4 years. Conversely, in a cohort of 830 participants, family history of CHD can predict the onset of ED (OR 1.75, CI: 1.17–2.61) after adjusting for age (24). Therefore, a possible bilateral association may exist between CVD and ED. However, previous studies mainly focused on the causal direction from ED to CHD. Our study reveals the causal links between CHD, heart failure and ED. Although most studies observed a significant association between CHD and ED, an insignificant relationship was reported. Kałka et al. (25) performed a cross-sectional study in Poland enrolling 751 men with coronary artery disease (CAD). They found that men with familial CAD have higher scores of International Index of Erectile Function 5 (IIEF-5) than those without. Their data did not support the risky role of CHD in the onset of ED. Besides, Speel et al. (8) also found that regardless of erectile function, participants aged 40–50 and 60–70 years have similar risks of CHD. The opposite findings may be attributed to the limited sample size and biases from observational designs, which is addressed using MR in this study.

Aside from CHD, the risky role of heart failure is also noted in our study. According to Apostolo et al. (26), 69.3% of heart failure patients had ED. In Turkey, Karabulut et al. (27) found that patients with heart failure had lower IIEF-5 scores than their counterpart without heart failure. This phenomenon was also replicated in rat models. Rodrigues et al. (28) reported that heart failure rats had lower intracavernosal pressure/mean arterial pressure than the normal controls, indicating impaired erectile function. This result may be attributed to increased ROCK 2 and MYPT-1 phosphorylation, leading to the contraction of cavernosal tissue and further ED. Our data further supports the causal role of heart failure in the pathogenesis of ED. However, the current evidence does not support the risky role of IHD in the occurrence of ED, as opposed to previous reports. The effect size is obvious (OR = 3.22), but not significant. The insignificant results may be attributed to the wide 95% confidence interval of calculated results. The insignificant association should be interpreted with caution and still needs further verification in future studies.

There are several merits and demerits of this study. The major strength is the MR design, clarifying the causal association between CVD and ED. The majority of previous studies are based on observational designs. Causal inference is challenging, which limits clinical decision-making. Additionally, this study examines several CVDs concurrently, providing more evidence between the cardiovascular system and erectile function. Prior studies mainly focused on one CVD. The major demerit is the usage of summary-level data, only providing binary variables. How CVD severity is correlated with ED severity remains unclear. Moreover, our findings are mainly obtained from cases of European descent Further verification in other races is necessary. In addition, the causal inference is based on the MR framework, requiring three preconditions that must be met. The genetic association may vary with sample descent and sample size. The causal conclusion must be interpreted with caution.

In conclusion, this study discloses that genetically predicted CHD and heart failure can causally increase the risk of ED, whereas atrial fibrillation, and IHD cannot. In clinical settings, CVD and ED should be evaluated simultaneously and doctors should provide corresponding treatments if necessary.

## 5. Conclusion

Using genetic data, this study revealed that genetically predicted CHD and heart failure may predict better ED compared with atrial fibrillation and IHD.

## Data availability statement

The original contributions presented in this study are included in the article/Supplementary material, further inquiries can be directed to the corresponding author.

## Ethics statement

Ethical review and approval can be accessed in the original studies and all the datasets used in this study are publicly accessible (https://gwas.mrcieu.ac.uk/). Informed consent was obtained from all subjects in the original genome-wide association studies.

## Author contributions

QYL: conceptualization. QYL and QL: data curation. QYL, QL, and BR: formal analysis. QYL and SB: writing—original draft and writing—review and editing. SB: supervision. All authors contributed to the article and approved the submitted version.

## Footnotes

1
http://www.nealelab.is/uk-biobank

## Supplementary material

Supplementary Figure 1 Funnel plots of cardiovascular diseases on ED. CHD, coronary heart disease; IVs, instrumental variables; IVW, inverse variance weighting.

Supplementary Table 1 Instrumental variables of coronary heart disease.

Supplementary Table 2 Instrumental variables of heart failure.

Supplementary Table 3 Instrumental variables of ischemic heart disease.

Supplementary Table 4 Instrumental variables of atrial fibrillation.
