# Supplementary figures and images for: Causal association between cardiovascular diseases and erectile dysfunction, a Mendelian randomization study
Source: Front Cardiovasc Med. 2023 Feb 9;10:1094330. doi: 10.3389/fcvm.2023.1094330 (PMC9947236; doi:10.3389/fcvm.2023.1094330)

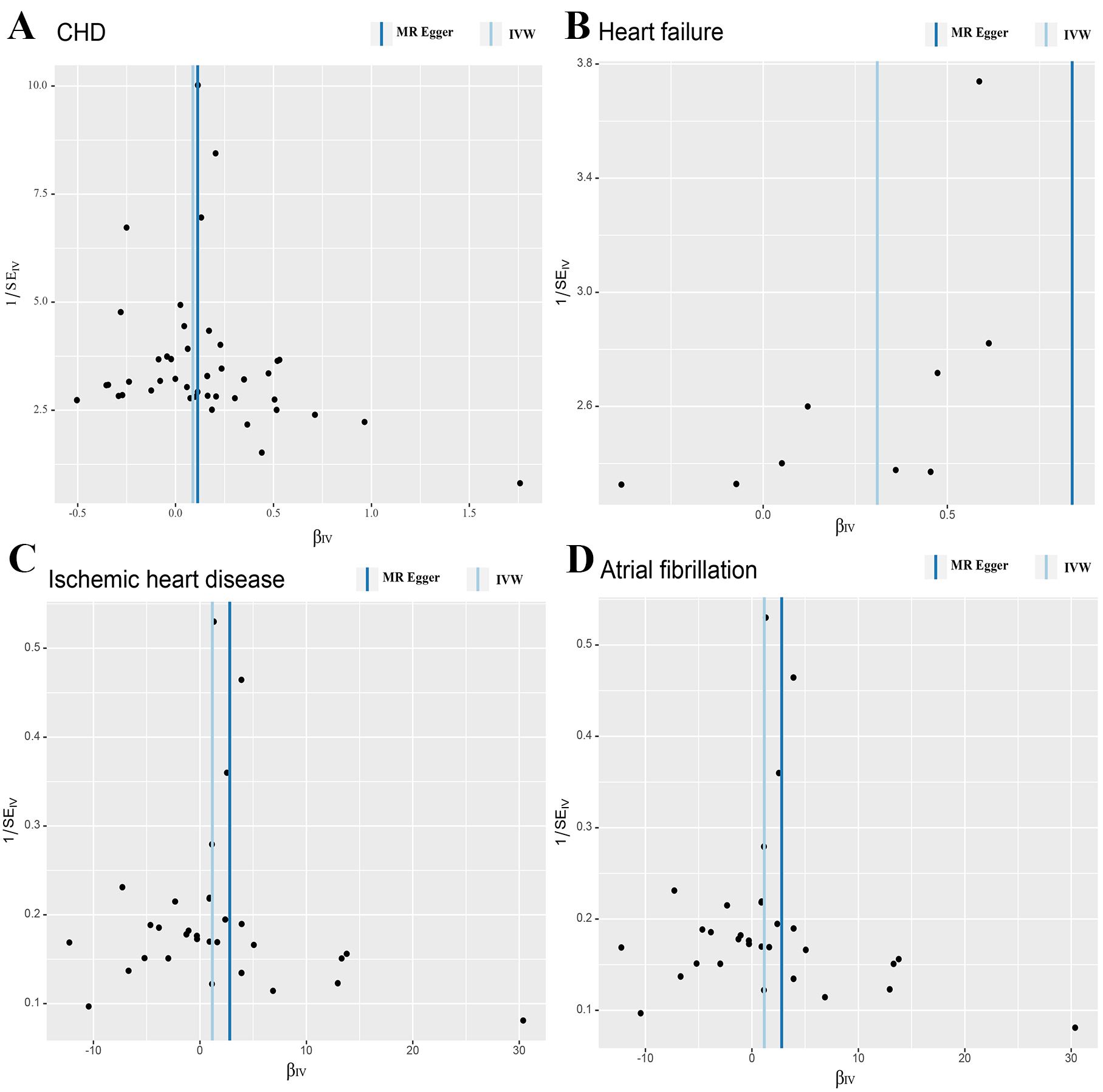

Supplement: Supplementary Figure 1 — Funnel plots of cardiovascular diseases on ED. CHD, coronary heart disease; IVs, instrumental variables; IVW, inverse variance weighting. [file Image_1.JPEG]
