# Supplementary material for: Causal association between cardiovascular diseases and erectile dysfunction, a Mendelian randomization study
Source: Front Cardiovasc Med. 2023 Feb 9;10:1094330. doi: 10.3389/fcvm.2023.1094330 (PMC9947236; doi:10.3389/fcvm.2023.1094330)
Supplement: Supplementary Table 1 — Instrumental variables of coronary heart disease. [file Table_1.DOCX]

| SNP | Chr | Position | A1 | A2 | Beta | EAF | P value | F |
| --- | --- | --- | --- | --- | --- | --- | --- | --- |
| rs10840293 | 11 | 9751196 | A | G | 0.0547 | 0.5498 | 1.28E-08 | 32.35 |
| rs11065979 | 12 | 1.12E+08 | T | C | 0.0686 | 0.3655 | 1.93E-10 | 40.54 |
| rs11191416 | 10 | 1.05E+08 | G | T | -0.0792 | 0.1275 | 4.65E-09 | 34.33 |
| rs11206510 | 1 | 55496039 | C | T | -0.0745 | 0.1524 | 2.34E-08 | 31.19 |
| rs11556924 | 7 | 1.3E+08 | T | C | -0.0726 | 0.3133 | 5.34E-11 | 43.05 |
| rs115654617 | 2 | 2.04E+08 | A | C | 0.1378 | 0.107 | 3.12E-18 | 75.81 |
| rs11617955 | 13 | 1.11E+08 | A | T | -0.0888 | 0.1064 | 3.55E-08 | 30.38 |
| rs11838776 | 13 | 1.11E+08 | A | G | 0.0686 | 0.2633 | 1.83E-10 | 40.64 |
| rs1199338 | 3 | 1.38E+08 | C | A | 0.0736 | 0.1619 | 3.90E-09 | 34.67 |
| rs12202017 | 6 | 1.34E+08 | G | A | -0.0668 | 0.3 | 1.98E-11 | 44.99 |
| rs1412444 | 10 | 91002927 | T | C | 0.0668 | 0.3691 | 5.15E-12 | 47.63 |
| rs16986953 | 2 | 19942473 | A | G | 0.0852 | 0.1047 | 1.45E-08 | 32.12 |
| rs17087335 | 4 | 57838583 | T | G | 0.0608 | 0.2146 | 4.59E-08 | 29.88 |
| rs17678683 | 2 | 1.45E+08 | G | T | 0.0988 | 0.0877 | 3.00E-09 | 35.18 |
| rs180803 | 22 | 24658858 | T | G | -0.1809 | 0.0293 | 1.64E-10 | 40.85 |
| rs186696265 | 6 | 1.61E+08 | T | C | 0.5504 | 0.0131 | 3.35E-30 | 130.4 |
| rs1870634 | 10 | 44480811 | G | T | 0.0759 | 0.6375 | 5.55E-15 | 61.05 |
| rs2107595 | 7 | 19049388 | A | G | 0.0734 | 0.2005 | 8.05E-11 | 42.25 |
| rs2128739 | 11 | 1.04E+08 | C | A | -0.0656 | 0.6765 | 7.05E-11 | 42.5 |
| rs2487928 | 10 | 30323892 | A | G | 0.0626 | 0.4182 | 4.41E-11 | 43.42 |
| rs2519093 | 9 | 1.36E+08 | T | C | 0.0797 | 0.1909 | 1.19E-11 | 45.99 |
| rs2681472 | 12 | 90008959 | G | A | 0.0741 | 0.2013 | 6.17E-11 | 42.77 |
| rs28451064 | 21 | 35593827 | A | G | 0.1276 | 0.1212 | 1.33E-15 | 63.95 |
| rs2891168 | 9 | 22098619 | G | A | 0.1934 | 0.4887 | 2.29E-98 | 443.1 |
| rs3918226 | 7 | 1.51E+08 | T | C | 0.1333 | 0.0645 | 1.69E-09 | 36.3 |
| rs41290120 | 19 | 45382675 | A | G | -0.181 | 0.035 | 1.37E-10 | 41.21 |
| rs4420638 | 19 | 45422946 | G | A | 0.0919 | 0.166 | 7.07E-11 | 42.5 |
| rs4468572 | 15 | 79124475 | C | T | 0.0772 | 0.5858 | 4.44E-16 | 65.71 |
| rs4593108 | 4 | 1.48E+08 | G | C | -0.0708 | 0.2047 | 8.82E-10 | 37.57 |
| rs4773141 | 13 | 1.11E+08 | G | C | 0.0697 | 0.3592 | 2.14E-09 | 35.84 |
| rs515135 | 2 | 21286057 | C | T | 0.0675 | 0.792 | 3.09E-08 | 30.65 |
| rs55730499 | 6 | 1.61E+08 | T | C | 0.3166 | 0.0562 | 5.39E-39 | 170.63 |
| rs56062135 | 15 | 67455630 | T | C | -0.0697 | 0.2057 | 4.52E-09 | 34.38 |
| rs56289821 | 19 | 11188247 | A | G | -0.1336 | 0.1004 | 4.44E-15 | 61.47 |
| rs56336142 | 6 | 39134099 | C | T | -0.0668 | 0.1927 | 1.85E-08 | 31.65 |
| rs663129 | 18 | 57838401 | A | G | 0.0582 | 0.2568 | 3.20E-08 | 30.58 |
| rs6689306 | 1 | 1.54E+08 | G | A | -0.056 | 0.5525 | 2.60E-09 | 35.46 |
| rs67180937 | 1 | 2.23E+08 | G | T | 0.0788 | 0.6631 | 1.01E-12 | 50.82 |
| rs7212798 | 17 | 59013488 | C | T | 0.08 | 0.1465 | 1.88E-08 | 31.61 |
| rs7528419 | 1 | 1.1E+08 | G | A | -0.1145 | 0.2142 | 1.97E-23 | 99.5 |
| rs8042271 | 15 | 89574218 | A | G | -0.0967 | 0.0977 | 3.68E-08 | 30.31 |
| rs9349379 | 6 | 12903957 | G | A | 0.1318 | 0.4316 | 1.81E-42 | 186.54 |
| rs9970807 | 1 | 56965664 | T | C | -0.1258 | 0.0849 | 5.00E-14 | 56.73 |
