# Supplementary material for: Causal association between cardiovascular diseases and erectile dysfunction, a Mendelian randomization study
Source: Front Cardiovasc Med. 2023 Feb 9;10:1094330. doi: 10.3389/fcvm.2023.1094330 (PMC9947236; doi:10.3389/fcvm.2023.1094330)
Supplement: Supplementary Table 2 — Instrumental variables of heart failure. [file Table_2.DOCX]

| SNP | Chr | Position | A1 | A2 | Beta | SE | P value | F |
| --- | --- | --- | --- | --- | --- | --- | --- | --- |
| rs11745324 | 5 | 1.37E+08 | A | G | -0.0528 | 0.0095 | 2.34E-08 | 30.89019 |
| rs17042102 | 4 | 1.12E+08 | A | G | 0.1103 | 0.0121 | 5.71E-20 | 83.09603 |
| rs17617337 | 10 | 1.21E+08 | T | C | -0.0561 | 0.0095 | 3.65E-09 | 34.87213 |
| rs4135240 | 6 | 36647680 | C | T | -0.0486 | 0.0084 | 6.84E-09 | 33.47449 |
| rs55730499 | 6 | 1.61E+08 | T | C | 0.1058 | 0.0157 | 1.83E-11 | 45.41215 |
| rs56094641 | 16 | 53806453 | G | A | 0.0454 | 0.008 | 1.21E-08 | 32.20563 |
| rs600038 | 9 | 1.36E+08 | C | T | 0.0569 | 0.0096 | 3.68E-09 | 35.13032 |
| rs660240 | 1 | 1.1E+08 | C | T | 0.0611 | 0.0097 | 3.25E-10 | 39.67701 |
| rs9295128 | 6 | 1.61E+08 | T | G | 0.2103 | 0.0338 | 4.96E-10 | 38.71196 |
