# Supplementary material for: Causal association between cardiovascular diseases and erectile dysfunction, a Mendelian randomization study
Source: Front Cardiovasc Med. 2023 Feb 9;10:1094330. doi: 10.3389/fcvm.2023.1094330 (PMC9947236; doi:10.3389/fcvm.2023.1094330)
Supplement: Supplementary Table 3 — Instrumental variables of ischemic heart disease. [file Table_3.DOCX]

| SNP | Chr | Position | A1 | A2 | Beta | EAF | P value | F |
| --- | --- | --- | --- | --- | --- | --- | --- | --- |
| rs10455872 | 6 | 1.61E+08 | G | A | 0.0174 | 0.0808s | 9.30E-70 | 311.84 |
| rs11591147 | 1 | 55505647 | T | G | -0.013 | 0.0177 | 1.52E-10 | 41.01 |
| rs11617955 | 13 | 1.11E+08 | A | T | -0.0053 | 0.1131 | 5.13E-10 | 38.63 |
| rs1199338 | 3 | 1.38E+08 | C | A | 0.0044 | 0.1613 | 1.83E-09 | 36.14 |
| rs12190287 | 6 | 1.34E+08 | G | C | -0.0037 | 0.3738 | 2.93E-11 | 44.23 |
| rs12324886 | 15 | 79054108 | G | A | -0.0044 | 0.7525 | 1.73E-12 | 49.77 |
| rs12369441 | 12 | 95536949 | C | T | -0.0062 | 0.0699 | 3.65E-09 | 34.8 |
| rs1333042 | 9 | 22103813 | G | A | 0.0102 | 0.4981 | 1.02E-80 | 362.2 |
| rs147932234 | 2 | 2.04E+08 | C | T | 0.0052 | 0.1276 | 1.27E-10 | 41.35 |
| rs16986953 | 2 | 19942473 | A | G | 0.006 | 0.068 | 2.92E-08 | 30.76 |
| rs186696265 | 6 | 1.61E+08 | T | C | 0.0285 | 0.0149 | 2.46E-37 | 163.07 |
| rs190712692 | 19 | 45425178 | A | G | -0.0077 | 0.0528 | 5.46E-10 | 38.51 |
| rs2011767 | 17 | 47340297 | T | C | -0.0037 | 0.4586 | 1.28E-11 | 45.85 |
| rs2107595 | 7 | 19049388 | A | G | 0.005 | 0.1515 | 1.90E-11 | 45.08 |
| rs2133189 | 1 | 2.23E+08 | T | C | 0.0037 | 0.7143 | 4.62E-10 | 38.83 |
| rs2839812 | 11 | 1.04E+08 | A | T | -0.0037 | 0.7208 | 9.98E-10 | 37.33 |
| rs28451064 | 21 | 35593827 | A | G | 0.0063 | 0.1318 | 1.15E-14 | 59.62 |
| rs2873195 | 17 | 2064702 | T | A | 0.0034 | 0.6816 | 3.42E-09 | 34.93 |
| rs35617716 | 8 | 19870263 | A | T | -0.0039 | 0.2613 | 3.71E-10 | 39.26 |
| rs3775059 | 4 | 96117230 | T | G | -0.0036 | 0.7893 | 3.99E-08 | 30.16 |
| rs429358 | 19 | 45411941 | C | T | 0.0059 | 0.1561 | 1.85E-15 | 63.22 |
| rs56125973 | 19 | 11188164 | C | T | -0.0058 | 0.1183 | 2.27E-12 | 49.24 |
| rs58721068 | 4 | 1.48E+08 | G | A | 0.0053 | 0.1427 | 7.38E-12 | 46.93 |
| rs6460891 | 7 | 12233919 | A | T | 0.0033 | 0.2663 | 4.88E-08 | 29.77 |
| rs660240 | 1 | 1.1E+08 | C | T | 0.0066 | 0.7844 | 1.08E-23 | 100.69 |
| rs72664318 | 1 | 56963627 | G | A | -0.0061 | 0.092 | 4.38E-11 | 43.44 |
| rs7500448 | 16 | 83045790 | G | A | -0.0039 | 0.2534 | 2.27E-10 | 40.22 |
| rs7668383 | 4 | 1.48E+08 | C | T | -0.0038 | 0.1841 | 4.13E-08 | 30.09 |
| rs78707197 | 6 | 82459034 | C | T | -0.0101 | 0.0219 | 3.63E-08 | 30.34 |
| rs8039305 | 15 | 91422543 | C | T | 0.0043 | 0.4759 | 2.54E-15 | 62.6 |
| rs9349379 | 6 | 12903957 | G | A | 0.0045 | 0.4055 | 1.28E-16 | 68.48 |
