# Supplementary material for: Causal association between cardiovascular diseases and erectile dysfunction, a Mendelian randomization study
Source: Front Cardiovasc Med. 2023 Feb 9;10:1094330. doi: 10.3389/fcvm.2023.1094330 (PMC9947236; doi:10.3389/fcvm.2023.1094330)
Supplement: Supplementary Table 4 — Instrumental variables of atrial fibrillation. [file Table_4.DOCX]

| SNP | Chr | Position | A1 | A2 | Beta | SE | EAF | P value | F |
| --- | --- | --- | --- | --- | --- | --- | --- | --- | --- |
| rs10213171 | 4 | 1.49E+08 | G | C | 0.091 | 0.0134 | 0.0609 | 1.32E-11 | 46.11829 |
| rs10458660 | 10 | 77936576 | G | A | 0.0537 | 0.0087 | 0.1726 | 6.78E-10 | 38.09869 |
| rs10520015 | 5 | 1.28E+08 | G | A | -0.0458 | 0.0082 | 0.2052 | 2.28E-08 | 31.19631 |
| rs10520260 | 4 | 1.74E+08 | G | A | -0.0457 | 0.0073 | 0.3214 | 3.36E-10 | 39.19103 |
| rs10741807 | 11 | 20011445 | C | T | -0.0729 | 0.0079 | 0.7551 | 1.59E-20 | 85.15318 |
| rs10753933 | 1 | 2.03E+08 | G | T | -0.0609 | 0.0067 | 0.5518 | 9.84E-20 | 82.61996 |
| rs10773657 | 12 | 1.23E+08 | A | C | -0.0575 | 0.0103 | 0.862 | 2.54E-08 | 31.16458 |
| rs10804493 | 3 | 1.12E+08 | A | G | 0.0558 | 0.007 | 0.6505 | 1.63E-15 | 63.54367 |
| rs10821415 | 9 | 97713459 | A | C | 0.0821 | 0.0067 | 0.4132 | 2.92E-34 | 150.1539 |
| rs10873298 | 14 | 77426525 | T | C | -0.0401 | 0.0069 | 0.6337 | 7.07E-09 | 33.77463 |
| rs11047497 | 12 | 24709133 | A | T | 0.0986 | 0.0159 | 0.046 | 5.62E-10 | 38.4556 |
| rs11102343 | 1 | 1.12E+08 | A | G | 0.0663 | 0.0111 | 0.0966 | 2.23E-09 | 35.67641 |
| rs11125871 | 2 | 61470126 | T | C | -0.0394 | 0.0068 | 0.3947 | 6.42E-09 | 33.5718 |
| rs11156751 | 14 | 32990437 | C | T | 0.0719 | 0.0077 | 0.2853 | 6.94E-21 | 87.19194 |
| rs11264280 | 1 | 1.55E+08 | T | C | 0.1347 | 0.0071 | 0.333 | 3.07E-79 | 359.9304 |
| rs11492754 | 10 | 1.05E+08 | C | T | -0.0549 | 0.0078 | 0.2648 | 1.99E-12 | 49.53994 |
| rs11598047 | 10 | 1.05E+08 | G | A | 0.1537 | 0.009 | 0.1621 | 8.95E-66 | 291.6505 |
| rs11658278 | 17 | 38031164 | C | T | -0.0443 | 0.0067 | 0.5208 | 3.46E-11 | 43.71775 |
| rs11773845 | 7 | 1.16E+08 | A | C | 0.1054 | 0.0067 | 0.5856 | 2.39E-55 | 247.4752 |
| rs117984853 | 6 | 1.49E+08 | T | G | 0.1228 | 0.012 | 0.1013 | 1.34E-24 | 104.7211 |
| rs1218578 | 1 | 1.55E+08 | G | A | -0.0434 | 0.0068 | 0.5607 | 1.48E-10 | 40.73443 |
| rs12188351 | 5 | 1.68E+08 | A | G | 0.0865 | 0.0145 | 0.0564 | 2.52E-09 | 35.5874 |
| rs12245149 | 10 | 65321147 | A | C | -0.047 | 0.0067 | 0.4739 | 1.66E-12 | 49.20918 |
| rs12260801 | 10 | 1.13E+08 | T | C | 0.0514 | 0.0093 | 0.1664 | 2.94E-08 | 30.54642 |
| rs12426679 | 12 | 76237987 | T | C | -0.0391 | 0.0067 | 0.5278 | 4.95E-09 | 34.05681 |
| rs12604076 | 17 | 76773638 | C | T | -0.0365 | 0.0066 | 0.5223 | 3.63E-08 | 30.58425 |
| rs1278493 | 3 | 1.36E+08 | A | G | -0.0389 | 0.0068 | 0.5645 | 8.77E-09 | 32.72513 |
| rs12809354 | 12 | 32978437 | C | T | 0.0718 | 0.0094 | 0.144 | 2.89E-14 | 58.34359 |
| rs12908004 | 15 | 80676925 | G | A | 0.0732 | 0.009 | 0.1637 | 4.12E-16 | 66.15111 |
| rs13195459 | 6 | 1.22E+08 | A | G | -0.0623 | 0.007 | 0.3615 | 4.15E-19 | 79.21 |
| rs133902 | 22 | 26164079 | T | C | 0.0419 | 0.0068 | 0.4268 | 9.14E-10 | 37.96734 |
| rs140185678 | 16 | 2003016 | A | G | 0.1659 | 0.0218 | 0.0351 | 2.43E-14 | 57.9135 |
| rs1458038 | 4 | 81164723 | T | C | 0.0434 | 0.0072 | 0.3087 | 1.74E-09 | 36.3341 |
| rs146518726 | 1 | 51535039 | A | G | 0.1605 | 0.0207 | 0.0328 | 8.27E-15 | 60.11867 |
| rs1545300 | 1 | 1.12E+08 | T | C | -0.0558 | 0.0073 | 0.3091 | 1.48E-14 | 58.42822 |
| rs1563304 | 17 | 44874453 | T | C | 0.0644 | 0.0092 | 0.178 | 2.56E-12 | 49 |
| rs17005647 | 3 | 69406181 | T | C | 0.0413 | 0.0069 | 0.3643 | 2.70E-09 | 35.8263 |
| rs17042059 | 4 | 1.12E+08 | A | G | 0.4029 | 0.0102 | 0.1076 | ####### | 1560.25 |
| rs17380837 | 12 | 26345526 | T | C | -0.0501 | 0.0072 | 0.307 | 4.80E-12 | 48.4184 |
| rs181926698 | 9 | 97003069 | T | C | 0.0718 | 0.0124 | 0.2637 | 6.20E-09 | 33.52784 |
| rs1957021 | 14 | 32924505 | C | T | 0.0583 | 0.008 | 0.2218 | 2.27E-13 | 53.10766 |
| rs2031522 | 6 | 87821501 | G | A | -0.0436 | 0.0068 | 0.3764 | 1.47E-10 | 41.11073 |
| rs2040862 | 5 | 1.37E+08 | T | C | 0.1084 | 0.0087 | 0.1775 | 1.08E-35 | 155.2459 |
| rs210628 | 6 | 1.18E+08 | C | G | -0.0434 | 0.0076 | 0.732 | 1.09E-08 | 32.61011 |
| rs2274115 | 9 | 1.39E+08 | G | A | 0.0487 | 0.0076 | 0.7003 | 1.69E-10 | 41.06111 |
| rs2288327 | 2 | 1.79E+08 | G | A | 0.0919 | 0.0089 | 0.1564 | 7.26E-25 | 106.623 |
| rs2359171 | 16 | 73053022 | A | T | 0.1746 | 0.0086 | 0.176 | 4.65E-91 | 412.1844 |
| rs2540949 | 2 | 65284231 | T | A | -0.0659 | 0.0068 | 0.3847 | 2.95E-22 | 93.9189 |
| rs2595117 | 4 | 1.12E+08 | T | C | -0.1422 | 0.0073 | 0.6993 | 1.26E-84 | 379.4491 |
| rs2738413 | 14 | 64679960 | G | A | -0.0778 | 0.0067 | 0.5049 | 2.55E-31 | 134.8372 |
| rs2759301 | 15 | 80994288 | A | G | 0.039 | 0.0067 | 0.4542 | 5.04E-09 | 33.88282 |
| rs2815301 | 16 | 2004718 | C | T | 0.0504 | 0.0085 | 0.7631 | 3.79E-09 | 35.15792 |
| rs2834618 | 21 | 36119111 | G | T | -0.0944 | 0.0112 | 0.1056 | 3.41E-17 | 71.04082 |
| rs28387148 | 2 | 1.27E+08 | T | C | 0.0741 | 0.0113 | 0.1051 | 6.25E-11 | 43.0011 |
| rs284277 | 1 | 10790797 | A | C | -0.0422 | 0.0069 | 0.6174 | 1.25E-09 | 37.40475 |
| rs2860482 | 12 | 57105938 | C | A | -0.054 | 0.0076 | 0.726 | 1.21E-12 | 50.48476 |
| rs28705758 | 4 | 1.11E+08 | A | G | 0.0708 | 0.0099 | 0.1253 | 7.91E-13 | 51.14417 |
| rs2885697 | 1 | 41544279 | T | G | -0.0439 | 0.007 | 0.6482 | 2.88E-10 | 39.33082 |
| rs297006 | 4 | 1.12E+08 | G | A | -0.0518 | 0.0071 | 0.6493 | 2.15E-13 | 53.22833 |
| rs3176326 | 6 | 36647289 | A | G | -0.0626 | 0.0085 | 0.1982 | 1.42E-13 | 54.23889 |
| rs337705 | 5 | 1.14E+08 | G | T | 0.0564 | 0.0068 | 0.3749 | 1.63E-16 | 68.79239 |
| rs34080181 | 3 | 66454191 | A | G | -0.0446 | 0.0069 | 0.379 | 1.28E-10 | 41.7803 |
| rs34969716 | 6 | 18210109 | A | G | 0.0702 | 0.0078 | 0.3051 | 1.60E-19 | 81 |
| rs35176054 | 10 | 1.05E+08 | A | T | 0.1391 | 0.01 | 0.1299 | 3.21E-44 | 193.4881 |
| rs35544454 | 2 | 2.13E+08 | T | A | -0.0589 | 0.0087 | 0.1918 | 1.10E-11 | 45.83446 |
| rs35569628 | 13 | 1.14E+08 | C | T | -0.0452 | 0.008 | 0.223 | 1.38E-08 | 31.9225 |
| rs35620480 | 8 | 11499908 | C | A | 0.054 | 0.0092 | 0.1567 | 5.15E-09 | 34.4518 |
| rs3781295 | 10 | 1.04E+08 | A | G | 0.0493 | 0.0068 | 0.3896 | 5.43E-13 | 52.5625 |
| rs3820888 | 2 | 2.01E+08 | C | T | 0.0684 | 0.0068 | 0.3921 | 5.75E-24 | 101.1799 |
| rs3951016 | 6 | 1.19E+08 | A | T | 0.0648 | 0.0067 | 0.459 | 2.15E-22 | 93.54065 |
| rs4073778 | 1 | 1.16E+08 | A | C | 0.0486 | 0.0067 | 0.5639 | 4.96E-13 | 52.61662 |
| rs422068 | 14 | 23864804 | C | T | 0.0439 | 0.007 | 0.3489 | 3.87E-10 | 39.33082 |
| rs464901 | 22 | 18597502 | C | T | -0.0508 | 0.0072 | 0.3353 | 1.53E-12 | 49.78086 |
| rs4871397 | 8 | 1.25E+08 | C | G | -0.0756 | 0.0138 | 0.9111 | 4.65E-08 | 30.01134 |
| rs4935786 | 11 | 1.22E+08 | A | T | -0.0463 | 0.0079 | 0.7327 | 4.85E-09 | 34.3485 |
| rs4963776 | 12 | 24779491 | T | G | -0.0913 | 0.0088 | 0.1821 | 1.84E-25 | 107.6406 |
| rs4965430 | 15 | 99268850 | G | C | -0.0441 | 0.0069 | 0.6136 | 1.26E-10 | 40.84877 |
| rs4999127 | 1 | 1.55E+08 | A | G | 0.0827 | 0.0098 | 0.839 | 4.28E-17 | 71.21293 |
| rs55734480 | 7 | 14372009 | A | G | 0.0548 | 0.0078 | 0.2494 | 2.20E-12 | 49.35963 |
| rs55754224 | 4 | 1.14E+08 | T | C | 0.0554 | 0.0075 | 0.2614 | 2.15E-13 | 54.56284 |
| rs55985730 | 7 | 1.28E+08 | G | T | 0.0867 | 0.0149 | 0.06 | 5.24E-09 | 33.85834 |
| rs56180201 | 5 | 1.73E+08 | T | G | -0.0468 | 0.0068 | 0.4123 | 8.09E-12 | 47.36678 |
| rs56181519 | 2 | 1.76E+08 | T | C | -0.0662 | 0.0077 | 0.2684 | 6.46E-18 | 73.91533 |
| rs56201652 | 7 | 92278116 | A | G | -0.0531 | 0.0075 | 0.267 | 1.74E-12 | 50.1264 |
| rs577676 | 1 | 1.71E+08 | T | C | -0.0923 | 0.0067 | 0.4383 | 1.62E-43 | 189.7815 |
| rs60212594 | 10 | 75414344 | C | G | -0.1176 | 0.0096 | 0.1438 | 9.20E-35 | 150.0625 |
| rs60902112 | 3 | 1.95E+08 | T | C | 0.0445 | 0.0079 | 0.2262 | 1.72E-08 | 31.72969 |
| rs62337205 | 4 | 1.12E+08 | G | A | -0.2015 | 0.0173 | 0.054 | 2.29E-31 | 135.6619 |
| rs62521286 | 8 | 1.25E+08 | G | A | 0.1202 | 0.0135 | 0.0663 | 4.50E-19 | 79.27594 |
| rs6462079 | 7 | 28415827 | A | G | 0.0466 | 0.0076 | 0.7208 | 8.79E-10 | 37.59626 |
| rs6480769 | 10 | 76851503 | T | A | -0.0387 | 0.0068 | 0.3992 | 1.15E-08 | 32.38949 |
| rs6560886 | 12 | 1.33E+08 | C | T | 0.051 | 0.009 | 0.7884 | 1.49E-08 | 32.11111 |
| rs6580277 | 5 | 1.43E+08 | G | A | 0.067 | 0.0079 | 0.2369 | 1.64E-17 | 71.92758 |
| rs6596717 | 5 | 1.06E+08 | A | C | -0.0404 | 0.0068 | 0.6049 | 3.00E-09 | 35.29758 |
| rs6689306 | 1 | 1.54E+08 | G | A | -0.046 | 0.0068 | 0.5872 | 1.36E-11 | 45.76125 |
| rs6747542 | 2 | 70106832 | C | T | -0.0554 | 0.0067 | 0.4642 | 1.10E-16 | 68.37068 |
| rs6771054 | 3 | 89489529 | C | T | -0.0457 | 0.0068 | 0.4035 | 2.42E-11 | 45.16631 |
| rs6790396 | 3 | 38771925 | G | C | 0.0627 | 0.0068 | 0.5959 | 2.40E-20 | 85.01925 |
| rs6818418 | 4 | 1.04E+08 | G | T | 0.0362 | 0.0066 | 0.4902 | 4.86E-08 | 30.08356 |
| rs6838973 | 4 | 1.12E+08 | T | C | -0.1514 | 0.0067 | 0.4406 | ####### | 510.6251 |
| rs6891790 | 5 | 1.73E+08 | T | G | -0.0729 | 0.0076 | 0.2828 | 4.53E-22 | 92.00848 |
| rs6994744 | 8 | 1.42E+08 | C | A | 0.0405 | 0.0066 | 0.4954 | 1.09E-09 | 37.65496 |
| rs7096385 | 10 | 69664881 | C | T | -0.0707 | 0.013 | 0.9078 | 4.87E-08 | 29.57686 |
| rs71454237 | 12 | 70013415 | A | G | -0.062 | 0.0084 | 0.209 | 1.78E-13 | 54.47846 |
| rs7170477 | 15 | 64103777 | A | G | 0.0393 | 0.0072 | 0.3041 | 4.98E-08 | 29.7934 |
| rs7225165 | 17 | 1309850 | A | G | -0.0655 | 0.0111 | 0.1133 | 3.20E-09 | 34.82063 |
| rs72700114 | 1 | 1.7E+08 | C | G | 0.2021 | 0.013 | 0.0756 | 3.29E-54 | 241.6829 |
| rs72700118 | 1 | 1.7E+08 | A | C | 0.1227 | 0.0101 | 0.1266 | 9.52E-34 | 147.5864 |
| rs72811294 | 17 | 12618680 | C | G | -0.072 | 0.0106 | 0.1131 | 9.67E-12 | 46.13742 |
| rs72926475 | 2 | 86594487 | A | G | -0.0683 | 0.0102 | 0.1228 | 2.37E-11 | 44.83747 |
| rs73041705 | 3 | 24463235 | C | T | -0.0443 | 0.0073 | 0.2985 | 1.55E-09 | 36.82661 |
| rs73241997 | 14 | 35173775 | T | C | 0.0733 | 0.0093 | 0.1424 | 2.94E-15 | 62.12152 |
| rs73366713 | 6 | 16415751 | A | G | -0.1035 | 0.0099 | 0.1396 | 1.53E-25 | 109.2975 |
| rs7373065 | 3 | 38710315 | C | T | -0.2024 | 0.0251 | 0.9508 | 7.58E-16 | 65.02398 |
| rs74022964 | 15 | 73677264 | T | C | 0.1132 | 0.009 | 0.157 | 3.51E-36 | 158.2005 |
| rs74500426 | 4 | 1.75E+08 | T | G | -0.0921 | 0.0127 | 0.0764 | 4.29E-13 | 52.59105 |
| rs74884082 | 14 | 73249419 | T | C | -0.0493 | 0.0078 | 0.2495 | 3.48E-10 | 39.94888 |
| rs74910854 | 7 | 74110705 | G | A | 0.09 | 0.0164 | 0.0693 | 4.31E-08 | 30.116 |
| rs7508 | 8 | 17913970 | A | G | 0.0711 | 0.0075 | 0.7109 | 1.69E-21 | 89.8704 |
| rs7529220 | 1 | 22282619 | C | T | 0.0621 | 0.0098 | 0.8469 | 1.98E-10 | 40.15421 |
| rs7578393 | 2 | 26165528 | T | C | 0.0614 | 0.0088 | 0.796 | 2.42E-12 | 48.68233 |
| rs76097649 | 11 | 1.29E+08 | A | G | 0.1151 | 0.0124 | 0.0933 | 1.26E-20 | 86.16031 |
| rs7612445 | 3 | 1.79E+08 | T | G | 0.0493 | 0.0084 | 0.1879 | 4.81E-09 | 34.44572 |
| rs7650482 | 3 | 12841804 | G | A | 0.0711 | 0.007 | 0.6401 | 1.79E-24 | 103.1676 |
| rs7723727 | 5 | 1.14E+08 | A | T | -0.0394 | 0.0066 | 0.5145 | 2.58E-09 | 35.63728 |
| rs77260060 | 10 | 1.05E+08 | A | G | 0.1062 | 0.0189 | 0.0369 | 1.85E-08 | 31.5737 |
| rs77316573 | 16 | 2265271 | T | C | 0.0529 | 0.0089 | 0.1991 | 3.26E-09 | 35.329 |
| rs775498 | 12 | 70071513 | G | A | 0.0423 | 0.0074 | 0.2798 | 1.05E-08 | 32.67513 |
| rs7789146 | 7 | 1.51E+08 | A | G | -0.0584 | 0.0087 | 0.1787 | 2.12E-11 | 45.05959 |
| rs7834729 | 8 | 21821778 | T | G | -0.0653 | 0.0104 | 0.1151 | 3.55E-10 | 39.42391 |
| rs79187193 | 1 | 1.47E+08 | A | G | -0.1162 | 0.0153 | 0.0569 | 3.15E-14 | 57.68055 |
| rs7980180 | 12 | 24727963 | A | G | 0.134 | 0.024 | 0.025 | 2.52E-08 | 31.17361 |
| rs80147385 | 1 | 1.7E+08 | T | C | -0.0886 | 0.0153 | 0.057 | 7.48E-09 | 33.53394 |
| rs8088085 | 18 | 48708548 | C | A | -0.0365 | 0.0067 | 0.4646 | 4.79E-08 | 29.6781 |
| rs883079 | 12 | 1.15E+08 | T | C | 0.0981 | 0.0074 | 0.7074 | 2.84E-40 | 175.7416 |
| rs9401427 | 6 | 1.22E+08 | A | C | -0.0662 | 0.0108 | 0.1071 | 8.92E-10 | 37.57236 |
| rs9506925 | 13 | 23368943 | T | C | 0.0449 | 0.0075 | 0.2669 | 2.72E-09 | 35.84018 |
| rs9899183 | 17 | 7452977 | T | C | 0.0452 | 0.0075 | 0.7138 | 2.02E-09 | 36.32071 |
| rs9953366 | 18 | 46474192 | C | T | 0.049 | 0.0073 | 0.6631 | 1.82E-11 | 45.05536 |
